# Effect of graphene-based intracanal medicament on push-out bond strength of different endodontic sealers in root canal dentin – an in vitro study

**DOI:** 10.2340/biid.v13.46558

**Published:** 2026-07-22

**Authors:** Rathna Piriyanga, Manish Ranjan, Anand Sherwood

**Affiliations:** a Department of Conservative Dentistry and Endodontics, Saveetha Dental College and Hospitals, Chennai, India; b Department of Conservative Dentistry and Endodontics, C.S.I. College of Dental Sciences and Research, Madurai, India

**Keywords:** graphene oxide, silver nanoparticles, allicin, intracanal medicament, push-out bond strength, endodontic sealer, root canal dentin

## Abstract

**Introduction:**

Graphene-based hydrogels incorporating antimicrobial agents, such as allicin and silver nanoparticles, offer potential therapeutic benefits. This study aimed to evaluate the effect of an allicin-incorporated graphene oxide–silver nanoparticle hydrogel on the push-out bond strength of endodontic sealers and the influence of different removal protocols.

**Materials and methods:**

A total of 60 extracted human single-rooted teeth were prepared to F3 size protaper and irrigated with 3% sodium hypochlorite and 17% Ethylenediaminetetraacetic Acid (EDTA) The experimental hydrogel (allicin + graphene oxide + silver nanoparticles in 2% sodium alginate base) was placed in all canals and incubated for 7 days. Specimens were randomised by sealer type (AH Plus, Mineral Trioxide Aggregate (MTA) Fillapex, Sealapex) and removal protocol: syringe irrigation, passive ultrasonic irrigation (PUI), or XP-Endo Finisher activation. Push-out bond strength was measured using a universal testing machine. A subset of specimens (*n* = 3 per protocol) underwent Scanning Electron Microscopy (SEM)/Energy Dispersive X-ray Spectroscopy (EDX) analysis to evaluate residual medicament. Data were statistically analysed.

**Results:**

Push-out bond strength was significantly influenced by both sealer type and removal protocol (*p* < 0.05). AH Plus exhibited the highest bond strength (mean ± standard deviation [SD]: 5.2 ± 1.4 MPa), followed by Sealapex (1.9 ± 0.3 MPa) and MTA Fillapex (1.5 ± 0.4 MPa). Activation-assisted protocols (PUI: 4.8 ± 1.2 MPa; XP-Endo Finisher: 5.0 ± 1.3 MPa) significantly increased bond strength compared to syringe irrigation alone (3.1 ± 1.0 MPa; *p* < 0.05). SEM/EDX analysis revealed minimal residual hydrogel after activation, without adversely affecting sealer adhesion. Bond strength was highest in the cervical third and lowest in the apical third (*p* < 0.05).

**Conclusion:**

The allicin-incorporated graphene oxide–silver nanoparticle hydrogel did not compromise the push-out bond strength of the evaluated endodontic sealers when appropriate medicament removal protocols were employed. PUI and XP-Endo Finisher activation effectively facilitated hydrogel removal and maintained sealer adhesion, supporting the potential use of this material as an intracanal medicament.

## Introduction

Successful endodontic treatment depends not only on effective elimination of intracanal microorganisms but also on the establishment of a durable seal between the obturating materials and root canal dentin [1]. The integrity of this sealer–dentin interface is critical for preventing microleakage, reinfection, and subsequent treatment failure [2]. Among the various factors influencing this interface, the condition of dentin at the time of obturation – particularly following the use of intracanal medicaments – has been shown to significantly affect the adhesion and retention of endodontic sealers [3, 4].

Intracanal medicaments are routinely employed between appointments to enhance microbial reduction within the root canal system, especially in cases with persistent or complex infections [5, 6]. Calcium hydroxide remains the most widely used medicament due to its antimicrobial properties and tissue compatibility; however, several studies have demonstrated that remnants of calcium hydroxide may adversely affect the bond strength of certain sealers, particularly epoxy resin-based formulations such as AH Plus [7, 8]. These observations highlight the importance of understanding how medicament composition and removal protocols influence dentin–sealer interaction.

Recent advances in nanotechnology have led to the development of novel graphene-based materials with promising antimicrobial and anti-inflammatory properties [9]. Graphene oxide and silver nanoparticle composites, particularly when combined with bioactive compounds such as allicin, have demonstrated enhanced antibacterial efficacy and favourable physicochemical characteristics in endodontic applications [10–12]. When formulated as hydrogels using biocompatible polymeric carriers such as sodium alginate, these materials offer improved handling, controlled placement, and intimate adaptation to canal walls, making them potential candidates for intracanal medicaments [13]. However, the presence of such hydrogels within the root canal raises important concerns regarding their retrievability and the potential influence of residual material on subsequent obturation procedures.

The removal of intracanal medicaments is typically achieved through adequate root canal instrumentation, followed by irrigation protocols involving sodium hypochlorite (NaOCl) and chelating agents, often supplemented by activation techniques such as passive ultrasonic irrigation (PUI) or non-enlarging agitation devices, which facilitate irrigant penetration and enhance the removal of residual medicament from the canal system [14–16]. Importantly, alterations to the canal surface resulting from medicament placement and subsequent removal procedures may influence dentin–sealer interactions and represent a clinically relevant scenario that warrants systematic investigation.

Push-out bond strength testing is a widely accepted method for assessing the adhesion of endodontic sealers to root canal dentin and provides insight into the interfacial integrity achieved under different clinical conditions [4]. The existing studies predominantly focus on conventional medicaments, with limited data evaluating the interaction between novel gel-based formulations, different sealer chemistries, and medicament removal techniques.

Therefore, the aim of the present in vitro study was to evaluate the effect of a novel allicin-incorporated graphene oxide–silver nanoparticle hydrogel intracanal medicament on the push-out bond strength of three commonly used endodontic sealers – MTA Fillapex, Sealapex, and AH Plus – following different medicament removal protocols. The study also compares the outcomes with those obtained using conventional intracanal medicaments and a no-medicament control. The null hypotheses tested were that the type of intracanal medicament would not influence the push-out bond strength of endodontic sealers, and the method of medicament removal would not affect sealer adhesion to root canal dentin.

## Materials and methods

This in vitro experimental study evaluated the effect of different intracanal medicaments and medicament removal protocols on the push-out bond strength of endodontic sealers to root canal dentin. The present investigation represents one arm of a broader research project evaluating the physicochemical and biological properties of a novel graphene-based intracanal medicament, approved by the Institutional Review Board (Approval No: SRB/SDC/PhD/ENDO-2364/24/TH-O14). Extracted human teeth were used in accordance with institutional and national ethical guidelines.

The study was designed and reported in accordance with the PRILE (Preferred Reporting Items for Laboratory studies in Endodontology) guidelines for in vitro endodontic research.

### Sample size calculation

Sample size was calculated based on previously published push-out bond strength studies, which commonly report a standard deviation (SD) of approximately 1.0 MPa for endodontic sealers. The minimum clinically relevant difference to be detected between groups was set at 1.0 MPa. Using an alpha level of 0.05 and a power of 80%, the required number of specimens was calculated using the following formula for comparison of two means:

$$n=\frac{{\left(Z_{\alpha /2}+Z_{\beta }\right)}^{2}\times 2\sigma^{2}}{d^{2}}$$Eq.1

Where:

- *Z_α_*^/2^ = 1.96
- *Z_β_* = 0.84
- *σ* = 1.0MPa
- *d* = 1.0MPa

This yielded a minimum of 16 slices per experimental group. Since multiple slices were obtained from each tooth and slices from the same tooth cannot be considered fully independent, a clustered design was adopted. To ensure adequate power while accounting for within-tooth correlation and potential specimen loss during sectioning or testing, 10 teeth per group were included, yielding up to 30 slices per group. Statistical analysis was adjusted accordingly using mixed-effects models.

### Specimen selection and storage

A total of extracted human single-rooted teeth (canines and premolars) with single canals were collected following extraction for orthodontic or periodontal reasons. Teeth with caries extending to the root, cracks, root resorption, previous endodontic treatment, calcified canals, or immature apices were excluded.

After debridement of soft tissue remnants, teeth were stored in 0.1% thymol solution at 4°C and used within 3 months of extraction to minimise alterations in dentin structure.

### Standardisation of specimens

Crowns were removed using a water-cooled diamond disc to obtain a standardised root length of 12 mm. Working length (WL) was established by introducing a size 10 K-file (Dentsply/Malliefer, Switzerland) into the canal until it was visible at the apical foramen and subtracting 1 mm.

### Root canal instrumentation and irrigation

All canals were prepared by a single operator using the ProTaper Universal rotary nickel–titanium system (Dentsply Maillefer, Ballaigues, Switzerland) to a standardised final apical preparation size of F3. During instrumentation, canals were irrigated with 2 mL of 3% NaOCl between files using a 27-gauge side-vented irrigation needle positioned 2 mm short of the WL.

Following instrumentation, a final irrigation protocol was performed using:

- 5 mL of 17% EDTA for 60 seconds to remove the smear layer,
- followed by 5 mL of sterile saline to neutralise irrigant remnants.

Canals were dried with sterile paper points.

### Intracanal medicaments

Specimens were randomly allocated to three medicament groups:

1. No medicament (negative control)
2. Calcium hydroxide paste (commercial product, manufacturer)
3. Allicin-incorporated graphene oxide–silver nanoparticle hydrogel formulated in a sodium-alginate base (test medicament)

The preparation and physicochemical characterisation of the graphene-based hydrogel have been previously described and validated in a separate published study. The same formulation was used in the present investigation without modification [12].

### Medicament placement and incubation

A standardised volume of medicament equivalent to 20 µL per millimetre of root length was introduced into the canal using a calibrated syringe and pre-bent NaviTip (Ultradent’s NaviTip™, USA) to ensure uniform distribution. The coronal access was sealed with temporary restorative material (3m Espe Cavit, Germany).

Specimens were stored in 100% humidity at 37°C for 7 days to simulate the clinical interappointment medicament period.

### Medicament removal protocols

After the incubation period, specimens in each medicament group were further randomised into three removal protocol subgroups:

R1 – Syringe irrigation

- 5 mL of 3% NaOCl (Prime Dental Products, India) for 60 seconds
- 5 mL of 17% EDTA (Anabond Desmear, India) for 60 seconds
- Final flush with 5 mL of 3% NaOCl

Irrigation was performed using a 27-gauge side-vented needle positioned 2 mm short of WL.

R2 – Passive ultrasonic irrigation

The same irrigants and volumes as R1 were used. Each irrigant was activated for 60 seconds using a non-cutting ultrasonic tip at low power, positioned 1 mm short of WL.

R3 – XP-Endo Finisher activation

The same irrigation regimen as R1 was used with mechanical agitation provided by an XP-Endo Finisher file operated according to the manufacturer’s instructions for 30–60 seconds in the presence of the irrigant, followed by a final NaOCl flush.

After completion of the removal protocol, canals were dried with paper points.

### SEM evaluation of canal walls (subset analysis)

SEM analysis was conducted exclusively for the experimental graphene-based intracanal medicament to qualitatively assess residual material on root canal dentin following different removal protocols. Three representative specimens were included for each removal protocol. After completion of the removal procedures, selected specimens were sectioned longitudinally along the long axis of the root. The samples were dehydrated, sputter-coated with gold, and examined using a scanning electron microscope. SEM images were obtained from representative regions of the cervical, middle, and apical thirds at standardised magnifications. The evaluation was limited to descriptive assessment of canal wall appearance and the presence or absence of residual medicament. No quantitative analysis or statistical comparison was performed.

### Obturation

Following medicament removal, canals were obturated using a single-cone technique with gutta-percha cones matched to the final preparation size and one of the following sealers:

- MTA Fillapex (Angelus, Londrina, Brazil)
- Sealapex (SybronEndo, Orange, USA)
- AH Plus (Dentsply Caulk, Milford, USA)

Sealers were mixed and applied according to the manufacturers’ instructions. The coronal access was sealed, and specimens were stored at 37°C and 100% humidity for 7 days to allow complete sealer setting.

### Sectioning of specimens

Each root was embedded in self-cure acrylic resin blocks to provide mechanical stability during sectioning. The embedded specimens were then sectioned perpendicular to their long axis using a hard-tissue microtome (Isomet1000; Buehler, USA) under continuous water cooling to obtain 1.0-mm-thick slices from the cervical, middle, and apical thirds. Slice thickness was verified using a digital caliper with an accuracy of 0.01 mm.

### Push-out bond strength testing

Push-out testing was performed using a universal testing machine (INSTRON 3369). Each slice was positioned on a metal support jig with an opening slightly larger than the canal diameter. Plunger tips of appropriate diameters (approximately 70% of the canal diameter) were selected to ensure contact only with the filling material.

A compressive load was applied in an apical-to-coronal direction at a crosshead speed of 0.5 mm/min until bond failure occurred. The maximum load at dislodgement (in Newtons) was recorded.

Bond strength values (MPa) were calculated using the following formula:


$$\text{Bond}\;\text{strength}\left(\text{MPa}\right)=\frac{F}{A}$$

Where *F*, is the maximum load (N) and *A*, is the bonded area, calculated as:

*A* = *π* × (*r*_1_+*r*_2_) × *h*

*r*_1_ and *r*_2_ represent the coronal and apical radii of the canal filling, and *h*, is the slice thickness.

### Statistical analysis

Data were analysed using statistical software (IBM SPSS STATISTICS 27). Normality of data distribution was assessed using the Shapiro–Wilk test. Push-out bond strength values were analysed using a mixed-effects linear model, with medicament type, sealer type, and removal protocol as fixed effects and tooth as a random effect to account for clustering of slices within teeth. Root third was included as a repeated measure.

Post-hoc pairwise comparisons were performed using Tukey’s adjustment for multiple testing. Statistical significance was set at *p* < 0.05.

## Results

### Push-out bond strength

Mean push-out bond strength values (MPa ± SD) for all sealers according to medicament type, removal protocol, and root canal level are presented in Tables 1–3.

**Table 1:** Push-out bond strength (MPa ± SD) of endodontic sealers – cervical third (*n* = 10 per group).

Medicament	Removal protocol	MTA Fillapex (*n* = 10)	Sealapex (*n* = 10)	AH Plus (*n* = 10)
None	-	1.30 ± 0.40^b^	1.10 ± 0.30^b^	5.10 ± 1.80^a^
Ca(OH)₂	Syringe	0.60 ± 0.25^c^	1.05 ± 0.30^bc^	3.30 ± 1.60^b^
Ca(OH)₂	PUI	0.95 ± 0.30^bc^	1.10 ± 0.35^b^	4.50 ± 1.40^ab^
Ca(OH)₂	XP-Endo Finisher	1.00 ± 0.35^bc^	1.15 ± 0.40^b^	4.60 ± 1.50^ab^
Hydrogel	Syringe	1.10 ± 0.38^b^	1.20 ± 0.32^b^	4.70 ± 1.60^a^
Hydrogel	PUI	1.25 ± 0.40^b^	1.25 ± 0.35^b^	5.00 ± 1.70^a^
Hydrogel	XP-Endo Finisher	1.28 ± 0.42^b^	1.30 ± 0.38^b^	5.05 ± 1.65^a^

Superscript letters indicate statistically significant differences within the Cervical third (*p* < 0.05, two-way Analysis of Variance [ANOVA] with Tukey post-hoc test). PUI: Passive ultrasonic irrigation; XP: XP-Endo Finisher; Ca(OH)₂: Calcium hydroxide.

**Table 2:** Push-out bond strength (MPa ± SD) of endodontic sealers – middle third (*n* = 10 per group).

Medicament	Removal protocol	MTA Fillapex (*n* = 10)	Sealapex (*n* = 10)	AH Plus (*n* = 10)
None	-	1.00 ± 0.30^b^	1.20 ± 0.35^b^	3.80 ± 1.50^a^
Ca(OH)₂	Syringe	0.50 ± 0.25^c^	1.15 ± 0.30^b^	3.20 ± 1.20^a^
Ca(OH)₂	PUI	0.80 ± 0.30^bc^	1.20 ± 0.35^b^	3.60 ± 1.30^a^
Ca(OH)₂	XP-Endo Finisher	0.85 ± 0.35^bc^	1.25 ± 0.40^b^	3.70 ± 1.35^a^
Hydrogel	Syringe	0.90 ± 0.32^bc^	1.25 ± 0.30^b^	3.60 ± 1.30^a^
Hydrogel	PUI	0.95 ± 0.30^b^	1.30 ± 0.35^b^	3.75 ± 1.40^a^
Hydrogel	XP-Endo Finisher	0.98 ± 0.33^b^	1.32 ± 0.38^b^	3.80 ± 1.45^a^

Superscript letters indicate statistically significant differences within the middle third (*p* < 0.05, two-way ANOVA with Tukey post-hoc test). PUI: Passive ultrasonic irrigation; XP: XP-Endo Finisher; Ca(OH)₂: Calcium hydroxide.

**Table 3:** Push-out bond strength (MPa ± SD) of endodontic sealers – apical third (*n* = 10 per group).

Medicament	Removal protocol	MTA Fillapex (*n* = 10)	Sealapex (*n* = 10)	AH Plus (*n* = 10)
None	-	1.20 ± 0.55^b^	1.35 ± 0.40^b^	4.80 ± 2.00^a^
Ca(OH)₂	Syringe	0.70 ± 0.30^c^	1.05 ± 0.25^bc^	4.40 ± 1.65^b^
Ca(OH)₂	PUI	0.90 ± 0.35^bc^	1.25 ± 0.35^b^	4.90 ± 1.80^ab^
Ca(OH)₂	XP-Endo Finisher	0.95 ± 0.40^bc^	1.30 ± 0.40^b^	5.00 ± 1.90^ab^
Hydrogel	Syringe	1.05 ± 0.45^b^	1.40 ± 0.35^b^	4.70 ± 1.75^a^
Hydrogel	PUI	1.15 ± 0.50^b^	1.45 ± 0.38^b^	4.90 ± 1.80^a^
Hydrogel	XP-Endo Finisher	1.18 ± 0.52^b^	1.48 ± 0.40^b^	5.00 ± 1.85^a^

Superscript letters indicate statistically significant differences within the apical third (*p* < 0.05, two-way ANOVA with Tukey post-hoc test). PUI: Passive ultrasonic irrigation; XP: XP-Endo Finisher; Ca(OH)₂: Calcium hydroxide.

### Overall statistical analysis

Mixed-effects analysis demonstrated significant main effects of sealer type (*p* < 0.001), medicament type (*p* = 0.003), removal protocol (*p* = 0.01), and root canal third (*p* = 0.02). A significant interaction was observed between medicament type and removal protocol (*p* = 0.02), whereas the three-way interaction among medicament type, sealer type, and removal protocol was not statistically significant (*p* > 0.05). AH Plus exhibited significantly higher push-out bond strength values than MTA Fillapex and Sealapex across all root thirds and removal protocols (*p* < 0.05).

### SEM evaluation of canal walls

**Figure 1:**
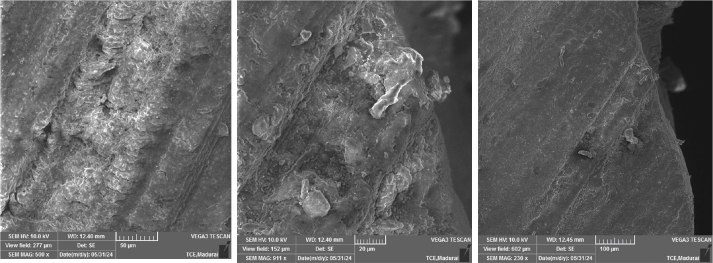
Representative SEM micrographs of canal walls following removal of the experimental hydrogel using Syringe irrigation, Passive ultrasonic irrigation (PUI) and XP-Endo Finisher activation.

Representative SEM micrographs of canal walls following removal of the experimental hydrogel are shown in Figure 1. Syringe irrigation demonstrated the presence of a thin residual film with scattered particulate deposits along the dentinal surface. In contrast, specimens treated with PUI or XP-Endo Finisher exhibited substantially cleaner canal walls, with minimal residual material observed.

### Effect of sealer type

Sealer type significantly influenced push-out bond strength (*p* < 0.001). AH Plus demonstrated significantly higher bond strength values than MTA Fillapex and Sealapex across all medicament groups, removal protocols, and root canal thirds (*p* < 0.05). Sealapex generally exhibited intermediate values, whereas MTA Fillapex showed the lowest bond strength values.

### Effect of calcium hydroxide medicament

In calcium hydroxide–treated specimens, syringe irrigation alone resulted in significantly reduced bond strength for AH Plus in the cervical and apical thirds compared with the no-medicament control (*p* < 0.05). Activation using either PUI or XP-Endo Finisher significantly improved push-out values compared with syringe irrigation (*p* < 0.05), though values remained slightly lower than the no-medicament control for AH Plus.

For MTA Fillapex and Sealapex, calcium hydroxide resulted in lower overall bond strengths, with no statistically significant differences between removal protocols (*p* > 0.05).

### Effect of experimental graphene-based hydrogel medicament

For specimens treated with the experimental allicin-incorporated graphene oxide–silver nanoparticle hydrogel, syringe irrigation resulted in slightly reduced push-out bond strength compared with the no-medicament control; however, this reduction was not statistically significant for any sealer or root third (*p* > 0.05).

When medicament removal was performed using PUI or XP-Endo Finisher, push-out bond strength values for all three sealers were statistically comparable to the no-medicament control at all root thirds (*p* > 0.05). No significant differences were observed between PUI and XP-Endo Finisher activation (*p* > 0.05).

AH Plus maintained the highest bond strength values in the hydrogel groups, followed by Sealapex and MTA Fillapex (Tables 1–3).

### Effect of root canal level

**Figure 2:**
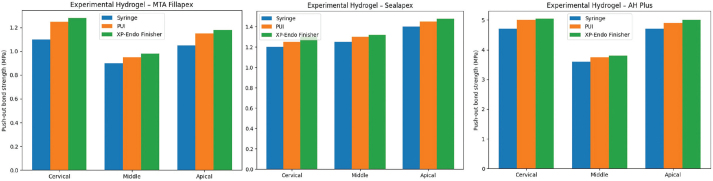
Graph shows push-out bond strength of different sealers following use of the experimental graphene-based hydrogel across different root thirds and removal protocols.

Across all groups, push-out bond strength values were highest in the cervical third, followed by the middle and apical thirds, irrespective of medicament or removal protocol (*p* < 0.05). Graph shows push-out bond strength of different sealers following use of the experimental graphene-based hydrogel across different root thirds and removal protocols (Figure 2).

## Discussion

This study was designed to investigate the effect of an allicin-incorporated graphene oxide–silver nanoparticle hydrogel on the push-out bond strength of commonly used endodontic sealers, with emphasis on removal efficiency, residual presence, and sealer compatibility. The findings demonstrate that the hydrogel can be effectively removed using activation protocols, and its application does not compromise adhesion of resin- or silicate-based sealers, thereby supporting its potential as a therapeutic intracanal medicament.

The experimental hydrogel, formulated in a non-crosslinked sodium alginate base, water-soluble nature facilitated removal using PUI and XP-Endo Finisher activation, achieving push-out bond strength values comparable to those of the no-medicament control across all root thirds and sealers. Even syringe irrigation alone left only minimal residual material, these remnants are unlikely to impair bonding; instead, they may confer prolonged antimicrobial activity, representing a unique advantage over traditional calcium hydroxide pastes, which can form mineralised residues that interfere with adhesion. This mechanistic insight reinforces the rationale for using nanoparticle-based hydrogels as intracanal medicaments.

Among the sealers evaluated, AH Plus exhibited the highest push-out bond strength in hydrogel-treated canals, consistent with its epoxy-resin chemistry and tubule-penetrating capability consistent with previous studies [17]. The hydrogel did not adversely affect AH Plus bonding, indicating that any residual GO/Ag particles or trace allicin remaining after medicament removal were compatible with resin infiltration and polymerisation under the conditions tested. Mechanistically, the epoxy–amine curing system of AH Plus is likely to tolerate sparse inorganic particulates. Previous biomaterials studies have reported that graphene oxide contains oxygen-containing functional groups, including hydroxyl, epoxy, and carboxyl groups, which may participate in interfacial interactions with polymeric matrices and contribute to improved dispersion and adhesion characteristics [18–21]. Therefore, any residual GO/Ag particles present after medicament removal are unlikely to adversely affect the sealer–dentin interface under the conditions evaluated in the present study. Also, Silver nanoparticles present at trace levels are chemically inert with respect to epoxy curing and therefore unlikely to inhibit cross-linking [22].

Allicin, as a small organosulfur molecule, may adsorb transiently to dentin surfaces or be present in minute amounts within the smear-like residual film; however, because polymerisation of epoxy resins is amine-driven rather than free-radical and because allicin concentrations after activation were minimal (as suggested by SEM/EDX], it is unlikely to have interfered with AH Plus setting or adhesion [23, 24]. Collectively, these factors explain why AH Plus retained superior bond strength in hydrogel-treated canals, while underscoring that effective medicament removal (activation-assisted irrigation) is essential to minimise residuals that could otherwise compromise sealer performance.

In contrast, MTA Fillapex and Sealapex, both calcium silicate–based sealers, demonstrated lower absolute push-out bond strength values compared with AH Plus, consistent with their hydration-dependent bonding mechanism rather than true chemical adhesion to dentin [25]. The hydrophilic, water-based nature of the sodium alginate–based graphene hydrogel is inherently compatible with the moisture-dependent setting reaction of silicate sealers, suggesting that the medicament itself does not chemically compromise sealer hydration or mineral interaction [26, 27]. However, adequate removal of the hydrogel remains essential to minimise physical interfacial remnants that could act as spacers at the sealer–dentin interface. The use of activation protocols ensured effective clearance of the hydrogel from canal walls, thereby allowing unhindered sealer adaptation and hydration.

In addition to sealer composition, the obturation technique may also influence the adaptation and interfacial behaviour of hydraulic and bioceramic sealers. Previous studies have demonstrated that different placement and compaction techniques can affect the marginal adaptation of calcium silicate–based sealers [28].

The study also highlights the importance of removal protocols for viscous or gel-based medicaments. While syringe irrigation alone left minimal residues, activation techniques significantly improved canal wall cleanliness, particularly in the apical third, where anatomical complexity often limits irrigant penetration consistent with previous researches [29]. This improvement translated into restoration of push-out bond strength to control levels. SEM images demonstrated that activated canals had markedly cleaner walls, with sparse GO/Ag particle deposition, supporting the push-out findings and confirming that activation-assisted removal is clinically advantageous [23]. These results underscore the need for careful consideration of medicament removal strategies in endodontic procedures to ensure optimal sealer adhesion, especially when using experimental or viscous materials.

The analysis of bond strength by root canal third revealed the expected gradient, with the highest values in the cervical third and lowest in the apical third, consistent with anatomical variations in dentin tubule density, orientation, and accessibility [30]. Importantly, the hydrogel did not exacerbate this gradient, and activation protocols effectively mitigated residual interference even in challenging apical regions, reinforcing the clinical feasibility of the hydrogel.

This study provides critical insights into the interplay between hydrogel properties, sealer chemistry, and removal efficacy. The hydrogel’s flowable, water-soluble composition allows effective canal filling while enabling efficient removal, balancing clinical utility with adhesion preservation. The trace presence of GO/Ag nanoparticles may offer prolonged antimicrobial activity; however, further investigations are required to confirm any such effects and determine their clinical significance [12, 31]. From a mechanistic perspective, these nanoparticles may remain adsorbed on dentin surfaces, providing a sustained therapeutic effect without negatively affecting sealer bonding. This unique combination of properties positions the hydrogel as a potentially superior alternative to traditional intracanal medicaments.

From a clinical perspective, the experimental hydrogel demonstrated favourable handling and removability characteristics, particularly when activation-assisted irrigation protocols were employed. Unlike calcium hydroxide, which may leave persistent residues that adversely affect sealer adhesion, the graphene-based hydrogel did not significantly compromise push-out bond strength following adequate removal [32]. The ability to maintain sealer adhesion while allowing effective retrieval from the canal system highlights its potential as a clinically useful intracanal medicament for contemporary endodontic practice.

Limitations include the qualitative nature of SEM/EDX analysis and the limited number of specimens evaluated, which precludes quantitative assessment of residual hydrogel. Additionally, in vitro push-out testing does not fully replicate the complex mechanical, biological, and thermal stresses of the oral environment, nor does it account for the long-term effects of residual nanoparticles on dentin or sealer stability.

Although graphene oxide and silver nanoparticles have demonstrated promising biomedical applications, their potential dose-dependent cytotoxic effects warrant further investigation. Therefore, additional studies evaluating the long-term biocompatibility, cytotoxicity, and tissue response of the present hydrogel formulation are required before clinical application. Future studies should investigate long-term bond durability, antimicrobial efficacy, and potential effects on dentin structure, especially under cyclic loading or in situ conditions.

## Conclusion

The allicin-incorporated graphene oxide–silver nanoparticle hydrogel demonstrates compatibility with resin- and silicate-based sealers and can be effectively removed using activation-assisted irrigation. Further studies are required to evaluate the biological significance of any residual nanoparticles and their potential antimicrobial effects. Within the limitations of this in vitro study, the material showed favourable interaction with endodontic sealers without adversely affecting push-out bond strength.

## Data Availability

The data that support the findings of this study are available from the corresponding author upon reasonable request.
